# Beyond Eschar: Unusual Skin Findings in Scrub Typhus

**DOI:** 10.4269/ajtmh.26-0240

**Published:** 2026-09-17

**Authors:** Tushar Biswas, Harpreet Singh, Atharva Pandharipande, Deba Prasad Dhibar, Manish Biswal, Aravind Sekar, Vikas Suri, Ashish Bhalla

**Affiliations:** ^1^ Department of Internal Medicine, Post Graduate Institute of Medical Education and Research, Chandigarh, India;; ^2^ Department of Microbiology, Post Graduate Institute of Medical Education and Research, Chandigarh, India;; ^3^ Department of Histopathology, Post Graduate Institute of Medical Education and Research, Chandigarh, India

A 22-year-old man hailing from Himachal Pradesh in North India was admitted with intermittent fever for 5 days, up to 38°C, associated with chills and rigors, along with a holocranial headache on a pain scale of 9/10, followed by altered sensorium in the form of irrelevant talk. He reported contact with vegetation while collecting feed for cattle. He also noticed multiple skin lesions over his arms, face, and legs, suggestive of palpable purpura with cutaneous infarction, as well as a blackish lesion over the nape of his neck. On examination, the patient was conscious and oriented with normal vitals and no focal neurological deficits. There was a black-colored necrotic lesion over the nape of his neck, suggestive of an eschar (Figure 1), with multiple cutaneous vasculitic lesions with skin peeling, suggestive of skin infarction, over his right anterior axillary wall, right lower limb, and left forearm (Figure 1). The possibility of tropical infection, most likely scrub typhus, was considered, and the patient was started on intravenous doxycycline (100 mg twice daily) and ceftriaxone (2 g twice daily). Bloodwork revealed thrombocytopenia (platelet count of 60,000 per cm^3^), with the rest of the hematological and biochemical profile being normal. Cerebrospinal fluid analysis revealed 19 cells with lymphocytosis and normal protein and sugar levels. A magnetic resonance image of the brain revealed mild leptomeningeal enhancement with multiple altered signal intensity lesions in the bilateral cerebral and cerebellar parenchyma, suggestive of encephalitis. Real-time polymerase chain reaction (PCR) testing targeting 47 kDa for *Orientia tsutsugamushi* in the blood yielded a positive result (cycle threshold value: 37.7). The 56 kDa result from the blood sample was negative. The blood culture result for this patient was also negative. His cerebrospinal fluid PCR test result for herpes simplex virus and cartridge-based nucleic acid amplification test (for *Mycobacterium tuberculosis*) was negative. A skin biopsy from the left forearm revealed panniculitis, endothelitis, and arteritis, consistent with scrub typhus infection (Figure 2). The patient responded to doxycycline (100 mg twice daily) for 10 days, with resolution of fever, skin lesions, and headache, and was discharged. Scrub typhus is a reemerging neglected infection caused by *Orientia tsutsugamushi* and transmitted by bites from trombiculid mite larvae. It is widely prevalent in the geographical area of the Tsutsugamushi Triangle. Clinical presentation ranges from acute febrile illness with generalized or regional lymphadenopathy (eschar site) to severe illness in the form of multiorgan dysfunction. The organism disseminates from the bite site to the lymph nodes and endothelial cells of various organs, such as the lungs, heart, kidneys, central nervous system, skin, and liver, resulting in endothelitis and vasculitis. Dermatological manifestations include an eschar at the bite site, a maculopapular rash, a vasculitic rash such as leucocytoclastic vasculitis, and purpura fulminans, as seen in the present case on the skin biopsy.^1^^–^^4^ Neurological manifestations include aseptic meningitis, meningoencephalitis, cerebral infarction, acute disseminated encephalomyelitis, transverse myelitis, and psychiatric manifestations. Mild-to-moderate elevation of cerebrospinal fluid protein (>50 mg%), lymphocytic pleocytosis, low-to-normal cerebrospinal fluid glucose, and no specific or diagnostic findings on radiology are common.^5^ The majority of patients respond to doxycycline, although those with severe multiorgan dysfunction require combination therapy with azithromycin.^6^^,^^7^

**Figure 1.**
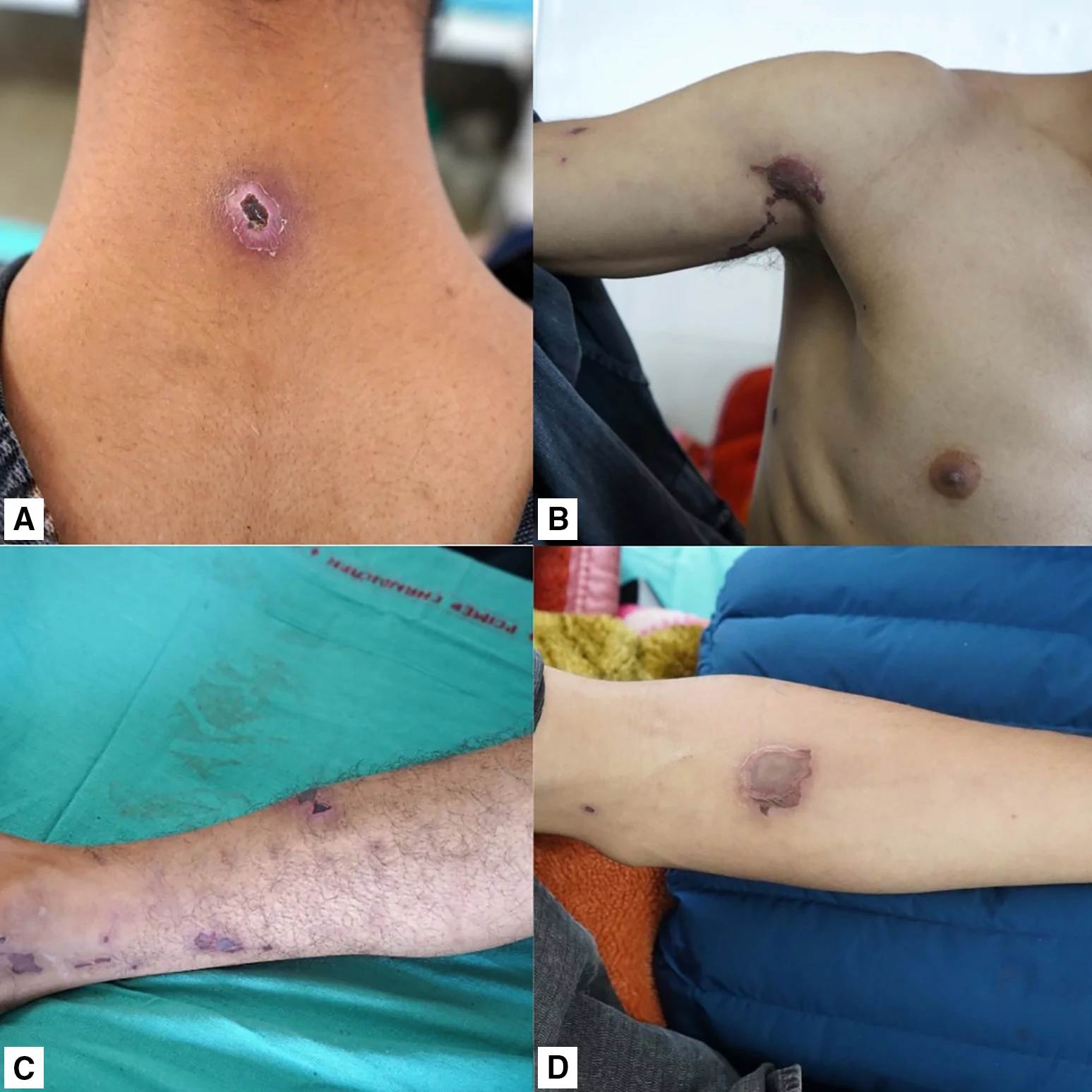
(**A**) Eschar at the nape of the neck. (**B**) Palpable purpuric lesions with cutaneous infarction on the anterior axillary wall, (**C**) right lower leg, and (**D**) left forearm.

**Figure 2.**
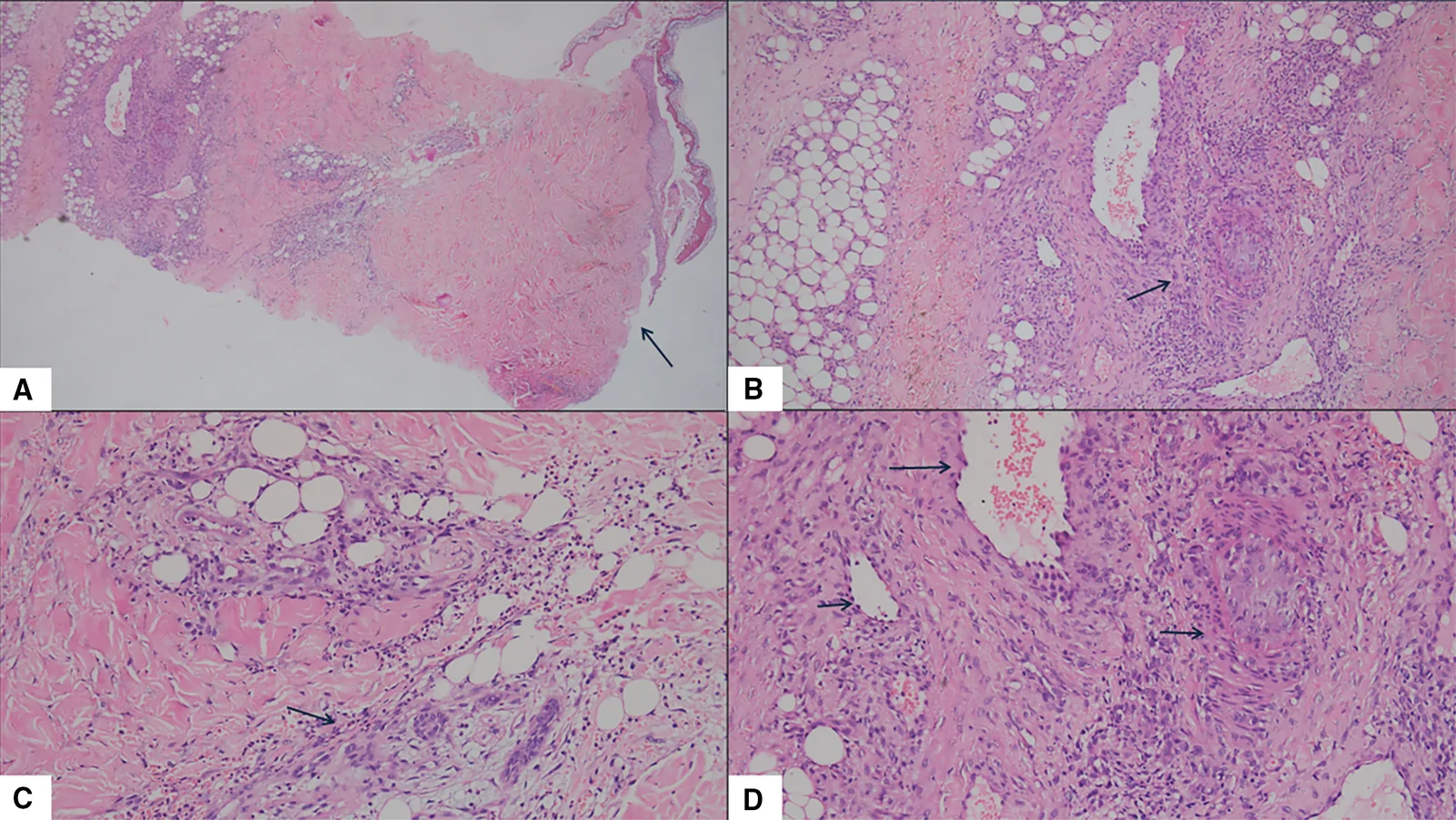
(**A**) Histopathology of skin lesions on the left forearm revealing focal epidermal necrosis at one edge of the biopsy specimen (hematoxylin and eosin [H&E], ×100). (**B**) There is a moderate mixed inflammatory cell infiltrate involving the dermis and extending into the subcutaneous tissue (H&E, ×200). (**C**) Higher magnification reveals predominant lobular inflammation with additional septal involvement, consistent with panniculitis (H&E, ×400). (**D**) The inflammatory infiltrate is also seen involving and infiltrating the arterial wall (H&E, ×400; arrow marks).
